# Comparative transcriptome analysis reveals new molecular pathways for cucumber genes related to sex determination

**DOI:** 10.1007/s00497-019-00362-z

**Published:** 2019-02-05

**Authors:** Magdalena Pawełkowicz, Leszek Pryszcz, Agnieszka Skarzyńska, Rafał K. Wóycicki, Kacper Posyniak, Jacek Rymuszka, Zbigniew Przybecki, Wojciech Pląder

**Affiliations:** 1 Department of Plant Genetics, Breeding and Biotechnology, Warsaw University of Life Sciences, Nowoursynowska 159, 02-776 Warsaw, Poland; 2 Laboratory of Zebrafish Developmental Genomics, International Institute of Molecular and Cell Biology, Ks. Trojdena 4, 02-109 Warsaw, Poland; 3 Present Address: Philip Morris International R&D, Philip Morris Products S.A., 2000 Neuchâtel, Switzerland

**Keywords:** Cucumber (*Cucumis sativus*), Sex determination, Flower development, Transcriptome profiling, Illumina sequencing

## Abstract

**Key message:**

Transcriptome data and qPCR analysis revealed new insight into genes regulatory mechanism related to cucumber sex determination.

**Abstract:**

Cucumber (*Cucumis sativus* L.) is an economically important crop cultivated worldwide. Enhancing the genomic resources for cucumber may enable the regulation of traits relevant to crop productivity and quality. Sequencing technologies and bioinformatics tools provide opportunities for the development of such resources. The aims of this study were to identify and characterize the genes involved in sex determination and flower morphogenesis in cucumber isogenic lines that differed regarding flower sex type. We obtained transcripts for 933 genes related to shoot apex development, among which 310 were differentially expressed genes (DEGs) among the male, female, and hermaphroditic lines. We performed gene ontology and molecular network analyses and explored the DEGs related to already known processes like: hormone synthesis and signaling, lipid and sugar metabolism; and also newly discovered processes related to cell wall, membrane, and cytoskeleton modifications; ion homeostasis which appears to be important for ethylene perception and signaling, and genes expression mediated by transcription factors related to floral organ identities. We proposed a new model of regulatory mechanism network of sex development in cucumber. Our results may be useful for clarifying the molecular genetics and the functional mechanisms underlying the sex determination processes.

**Electronic supplementary material:**

The online version of this article (10.1007/s00497-019-00362-z) contains supplementary material, which is available to authorized users.

## Introduction

Cucumber (*Cucumis sativus*), which belongs to the family *Cucurbitaceae*, is an economically and nutritionally important vegetable crop cultivated worldwide. Cucumber yield depends not only on favorable growth conditions, but also on efficient pollination as well as the type and quantity of flowers that develop on plants (Pawełkowicz et al. 2016a). The diverse cucumber flowers can be divided into the following three main types: staminate (male), pistillate (female), and hermaphroditic (both male and female organs). Plants in the family *Cucurbitaceae* fail to produce fruit when pollination does not occur with the exception of hybrid variants only with female flowers. Cucumbers usually have female and male flowers on the same plant, meaning that they are self-pollinating and do not have to receive pollen from other plants. In hybrid varieties, the number of female flowers on the plant is enhanced in order to increase the yield of fruits, but here pollination is complicated. Bumblebees and honeybees, which are the most effective cucumber pollinators, transfer pollen from male to female flowers, ultimately resulting in the production of fruits. In rare cases, flowers are manually pollinated. Few commercial cucumber (hybrid varieties) mostly those grown in a greenhouse, could produce fruit in the absence of pollination, so-called parthenocarpic (seedless); this necessitates the growth of gynoecious plants with only female flowers. Although such plants do not produce pollen of their own, they can be still fertilized by other varieties of cucumbers by bees. Fruits resulting from pollination will be often deformed.

On the basis of the position and occurrence of different flower types on the stem, plants can be divided into the following types: monoecious (male and female flowers), gynoecious (only female flowers), andromonoecious (hermaphroditic and male flowers), hermaphroditic (only hermaphroditic flowers), trimonoecious (female, male, and hermaphroditic flowers), and androecious (only male flowers). Beside the lack of ovary, male flowers differ from female flowers regarding appearance also by having shorter stem. Moreover, the female flower produces an ovary in a small fruit shape, at the base of the stem. Hermaphroditic flowers consist of a round and short ovary, and fruits derived from these flowers are formless and have no commercial value. Elucidating the mechanism underlying flower development may generate new information regarding regulatory activities relevant to manipulating flower sex determination in cucumber as well as in other species. Although sex determination is one of the key developmental processes during plant sexual reproduction, it remains poorly understood.

Several studies have attempted to characterize the molecular aspects of cucumber sex determination. During the early cucumber flower development stages, floral primordia are bisexual and contain the initial forms of anthers and pistils. In this species, sex determination requires the selective arrested development of the staminate or pistillate primordia (Bai et al. 2004). Cucumber sex expression is mainly determined by the *F*, *m*, *a*, *gy*, and *h* genes. The female (*F*) gene encodes a 1-aminocyclopropane-1-carboxylic acid synthase (CsACS1G) that regulates the number of female flowers (Mibus and Tatlioglu 2004; Trebitsh et al. 1997). The andromonoecious gene (*M*) also encodes a putative 1-aminocyclopropane-1-carboxylic acid synthase (CsACS2), while the *m* allele is mutated at a conserved site (resulting in the Gly33Cys amino acid change). The mutated enzyme exhibits decreased activity (Boualem et al. 2008; Li et al. 2009). The *F* gene promotes femaleness, while the *m* gene regulates the appearance of hermaphroditic flowers on the plant. Both genes are involved in ethylene synthesis and considerably affect sex determination by enhancing femaleness (Li et al. 2012). Sex expression can be also influenced by many environmental factors, including the photoperiod, temperature, and exposure to plant hormones (e.g., auxin and gibberellins) (Malepszy and Niemirowicz-Szczytt 1991; Perl-Treves 1999; Yamasaki et al. 2005). The *CsACS2* expression level is correlated with ethylene production, and may induce the expression of *CsACS* genes, suggesting there is positive feedback (Li et al. 2012). There are relatively few reports describing *A/a*, *Gy/gy*, and *H*/*h* genes. According to a recent report, the *a* gene may be related to *CsACS11*, which encodes another ACS protein in the ethylene biosynthesis pathway (Boualem et al. 2015). The earlier studies showed that the *a* gene may be associated with *CsRAN1*, which encodes a copper transporter (Terefe 2005). On the basis of sex determination in *Cucumis melo*, *gy* can be assumed to be related to *CsWIP* (Boualem et al. 2015), which encodes a transcription factor with zinc finger domains and could be related to a serine/threonine kinase gene, *CsPSTK1* (Pawełkowicz et al. 2012). Even though the sex expression is extensively studied (Przybecki et al. 2003, 2004; Wu et al. 2010), there is still very little information about the genes responsible for sex determination in cucumber.

Characterizing the processes leading to flower sex determination has great practical applications, as the sex of a flower or plant often limits how the plant is bred and cultivated. Herein, we present our transcriptome data concerning the shoot apex and leaves of cucumber lines that differ regarding sex. Furthermore, genes differentially expressed among the male, female, and hermaphroditic flowers were investigated to reveal differences in the regulation of key pathways. We proposed for the first time regulatory networks of proteins which are related to sex determination, and based on the functional annotation, we propose the new hypothetical scheme presenting the new processes with identified herein factors related to flower sex formation.

Our data represent an important genetic resource for clarifying the molecular mechanisms regulating cucumber sex determination and flower morphogenesis. The results of this study may be useful for future gene expression and functional genomic studies as well as cucumber breeding.

## Materials and methods

### Plant materials and RNA isolation

The following six cucumber lines that varied regarding flower sex type were taken for RNA sequencing (RNA-seq) analysis: two male samples (B10 and 859), two female samples (2gg and Gy3), and two hermaphroditic samples (2667 and Hgy3). Plants were cultivated in plastic pots in a greenhouse with a 16-h light (25–27 °C)/8-h dark (18–20 °C) photoperiod. The light intensity (i.e., photosynthetically active radiation) in the greenhouse was 1500 µmol m^−2^ s^−1^. For a subsequent RNA isolation, leaves and the shoot apex (with newly formed, < 1 mm long floral buds) were collected, frozen in liquid nitrogen, and stored at − 80 °C. Total RNA extracted from the leaves of six lines was pooled to form a representative sample of vegetative organs for a transcriptome analysis. The RNA extracted from the shoot apex of each line was analyzed separately. Total RNA was extracted using the TRIzol reagent (Invitrogen, Waltham, MA, USA). The concentration (optical density 260 nm/280 nm ratio) and quality (optical density 260 nm/230 nm ratio) of the extracted RNA were determined with the NanoDrop 2000 spectrophotometer (Thermo Fisher Scientific, Waltham, MA, USA). The RNA concentration was adjusted to 100 ng µL^−1^. For the quantitative real-time polymerase chain reaction (qPCR) analysis, RNA was treated with DNaseI from the TURBO DNA-free kit (Ambion, Austin, TX, USA) and then checked by PCR to ensure that there was no contaminating DNA. First-strand cDNA was synthesized using the High-Capacity cDNA Reverse Transcription Kit (Thermo Fisher Scientific, Waltham, MA, USA).

### RNA sequencing library construction and Illumina sequencing

Approximately 5–10 μg total RNA was used as the template for constructing RNA-seq libraries. The subsequent purification of polyadenylated RNA as well as the RNA fragmentation, cDNA synthesis, and PCR amplification were completed according to the Illumina RNA-seq protocol (Illumina, Inc., San Diego, CA, USA).Two replicas of each sex-specific library from shoot apex and leaves were sequenced using an Illumina Genome Analyzer at the Aqencourt–Beckman Coulter Genomics, USA. As a technical control of the experiment, the internal standard was used according to the company’s procedures. Single-end sequence reads (35–40 bp) were generated, and the RNA-seq read quality was evaluated based on the Illumina purity filter, percentage of low-quality reads, and the distribution of phred-like scores during each cycle. The data presented herein passed the quality control filtering based on these metrics. Sequences are available in the Sequence Read Archive of the National Center for Biotechnology Information (BioProject PRJNA359788).

### Gene annotation

*Cucumis sativus* L. genes were annotated using the BLAST + 2.2.26 program with the following parameters: blastp -F “mS” -b 1500 -v 1500 -e 0.001 -M BLOSUM45, and further filtered with an e-value cutoff of 1e-03 and the InterProScan (version 5) program (Jones et al. 2014). The unigenes were validated and annotated by aligning them to sequences from the following databases: Phytozome (Goodstein et al. 2012), PANTHER (protein analysis through evolutionary relationships) (Mi et al. 2013), Eukaryotic Orthologous Groups (KOG) (Tatusov et al. 2003), Kyoto Encyclopedia of Genes and Genomes (KEGG) (Kanehisa and Goto 2000), and The Arabidopsis Information Resource (TAIR) (Lamesch et al. 2011). Additionally, we annotated all proteins with protein domains from the Pfam-A (version 26) database (Finn et al. 2009) using the HMMER (version 3.0) program (Mistry et al. 2013) and an e-value cutoff of 1e−05. Finally, genes were annotated with gene ontology (GO) terms for *Arabidopsis thaliana* orthologs (Lamesch et al. 2011) and with using Blast2GO software (Conesa and Götz 2008).

### RNA sequencing data analysis

The *C. sativus* genome sequence and annotated genes were retrieved from the Phytozome (version 10) database (Goodstein et al. 2012). Sequence reads for each tissue sample were mapped to the reference genome (Gy14) using TopHat (version 2.0.12) (Kim et al. 2013), which is a splice-aware, short-read aligner. Transcript levels were subsequently quantified using the Flux Capacitor (version 1.6.1) program (Montgomery et al. 2010).

### Identification of differentially expressed genes

Transcriptomes for the shoot apex and leaves were compared to analyze gene expression patterns. Transcripts that were expressed only in the shoot apex were compared to identify genes specifically involved in generative organ development and expressed in the third and fourth whorls of floral buds. Differentially expressed genes (DEGs) were detected using DESeq (Anders and Huber 2010), with an adjusted *p* value cutoff of 0.05 and a fold change (FC) ≥ 4 as the threshold. We defined genes with a normalized expression level below 10 as not expressed. We completed the following three comparisons: female *vs* male; female *vs* hermaphrodite; and male *vs* hermaphrodite. A cluster analysis was performed with the MeV 4.9.0 program (http://mev-tm4.sourceforge.net/svnroot/mev-tm4/trunk) using Pearson correlation.

### Quantitative real-time PCR

We designed qPCR primers for specific genes using the Primer3 (version 2.3.6) software and the *C. sativus* Gy14 (version 1.0) sequence. Reference genes were chosen based on the experimental results as previously described by Skarzyńska et al. (2016). Using the geNorm applet (Vandesompele et al. 2002), we selected two of the five tested reference genes for further analyses. Details regarding the qPCR primers and the corresponding amplicon lengths are provided in Table S1.

A qPCR assay was completed to verify RNA-seq data and compare transcript levels among the analyzed cucumber lines with different flower sex types. Of the genes identified by the bioinformatics analysis as being differentially expressed among the cucumber lines, 16 were analyzed by qPCR. All qPCR assays were completed using three biological replicates, with three technical replicates each. The cDNA included in the qPCR was reverse transcribed from 1 µg total RNA and diluted (1:5). The qPCR assays were completed with 4 µL cDNA, the Power SYBR^®^ Green PCR Master Mix (Thermo Fisher Scientific, Waltham, MA, USA), and the Applied Biosystems 7500 Real-Time PCR System (Thermo Fisher Scientific, Waltham, MA, USA). A melting curve analysis was completed immediately after the qPCR. The mean amplification efficiency was calculated based on the linear regression for the slope of the regression line in the exponential phase with LinRegPCR (version 2015.3) (Ramakers et al. 2003). Relative expression levels were determined according to the 2^−ΔΔCt^ method with RStudio and EasyqPCR from the Bioconductor software package (Pape 2012).

### Functional annotation and molecular networks

A total of 310 DEGs were analyzed using MapMan 3.5.1 (Usadel et al. 2009). The cucumber mapping file was derived from the Phytozome (version 10) database. The STRING algorithm (version 10.5) (Szklarczyk et al. 2017), with *A. thaliana* as a model, was applied for an additional analysis of the possible interactions between the selected proteins. The confidence cutoff for the in silico-predicted molecular network was set at 0.70. Furthermore, we used the Cytoscape stringAPP (version 3.4.0) to edit the layout of our map (Shannon et al. 2003). Nodes were grouped together with their neighbors based on their clustering coefficient for greater output clarity. Additionally, each node was color-coded based on the flower sex type in which the gene was most highly expressed (i.e., male, female, or hermaphrodite).

## Results and discussion

**Fig. 1:**
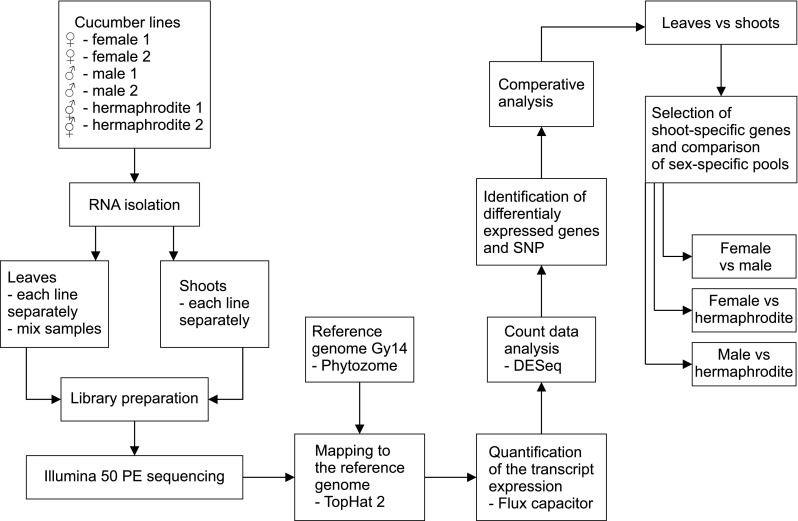
Overview of the transcriptome comparisons

To identify the genes responsible for early flower sex determination, we performed RNA-seq analysis. The sequenced transcriptomes were compared to identify DEGs associated with the formation of male, female, and hermaphroditic flowers. First, we compared leaf and shoot apex transcriptomes to distinguish between the vegetative organ- and generative organ-specific transcripts. Second, we compared the transcriptomes for the generative organs from the shoot apex to reveal the genes specifically expressed in different flower sex types. The general overview of our transcriptome analyses is presented in Fig. 1.

### Sequencing, mapping, and gene annotation

Short reads were aligned to the *C. sativus* Gy14 reference genome. We observed that 89.51–94.5% of the reads were aligned to the genome, of which 84.24–92.24% of the reads were uniquely aligned (Table 1). These results suggested that high-quality sequencing libraries had been prepared. We observed that 29.5–48.6 Mb (15–24%) of the *C. sativus* genome was expressed.

**Table 1:** Mapping statistics for the short reads aligned to the reference genome

Sample	Reads	Aligned	(%)	Aligned uniquely	(%)	Concordant pairs	(%)
Leaves mix 1	174,302,526	156,053,692	89.53	146,824,160	84.24	67,377,737	77.31
Leaves mix 2	100,610,456	90,830,182	90.28	85,189,347	84.67	39,376,061	78.27
Male 1	176,901,600	166,438,176	94.09	162,745,831	92.00	78,897,532	89.20
Male 2	161,712,846	148,354,681	91.74	143,956,991	89.02	67,078,816	82.96
Female 1	156,338,944	145,721,071	93.21	142,613,880	91.22	68,190,588	87.23
Female 2	159,498,702	150,720,535	94.50	147,119,433	92.24	70,972,223	88.99
Hermaphrodite 1	148,861,648	134,632,599	90.44	130,806,274	87.87	59,962,921	80.56
Hermaphrodite 2	140,273,124	129,676,914	92.45	126,846,024	90.43	59,289,428	84.53
Control	142,829,162	520	0.00	393	0.00	26	0.00

The unigenes were validated and annotated by aligning them to sequences from the following databases: Phytozome, InterProScan, Pfam, PANTHER, KOG, KEGG, and TAIR (Table 2, Table S2). A total of 30,364 mapped unigenes were matched to known genes in databases. Additionally, 25,628 *C. sativus* genes were annotated using InterProScan to obtain protein potential functions.

**Table 2:** Details regarding unigene annotations based on public databases

Public databases	Number of unigenes	Percentage of unigenes (%)
Phytozome	30,364	100
InterProScan	25,628	84.4
PFAM	23,605	77.7
Panther	19,700	64.8
KOG	13,648	44.9
KEGG	5130	16.8
GO	17,924	59.1
TAIR	28,383	93.4

### Identification of differentially expressed genes

**Fig. 2:**
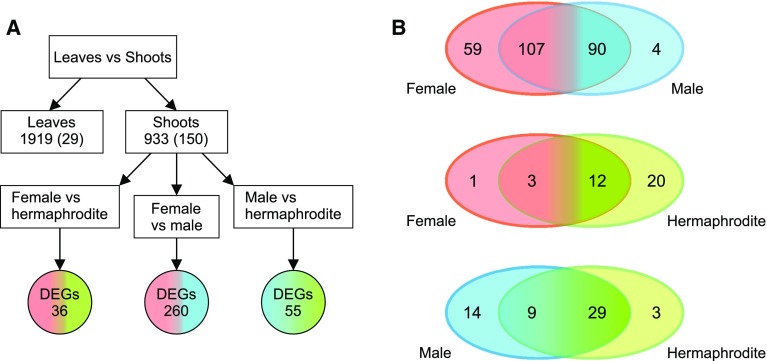
Schematic representation of the completed analysis. **a** Comparison of leaf and shoot apex revealed genes with changed expression in vegetative and generative tissues. The number of genes exclusively expressed in sample was shown in brackets. The 933 DEGs that exhibited upregulated expression in tissues with small floral buds and generative organs were further analyzed to detect genes differentially expressed among female, male, and hermaphroditic lines. **b** Venn diagrams of 310 sex-specific DEGs (in three comparisons: female vs male, female vs hermaphrodite, and male vs hermaphrodite) show number of genes with higher or exclusive expression in female, male or hermaphroditic line in each comparison

We analyzed DEGs in four ways. First, we separated vegetative organ- and generative organ-specific transcripts by comparing the leaf and shoot apex transcriptomes. This comparison yielded 2852 DEGs (false discovery rate < 0.05; fold change: 4), of which 933 were more highly expressed in the shoot apex and 150 of them were not expressed in vegetative tissue (i.e., shoot specific). Additionally, 1919 genes were more highly expressed in leaves, including 29 genes that were not expressed in the shoots (Fig. 2; Table S3).

**Fig. 3:**
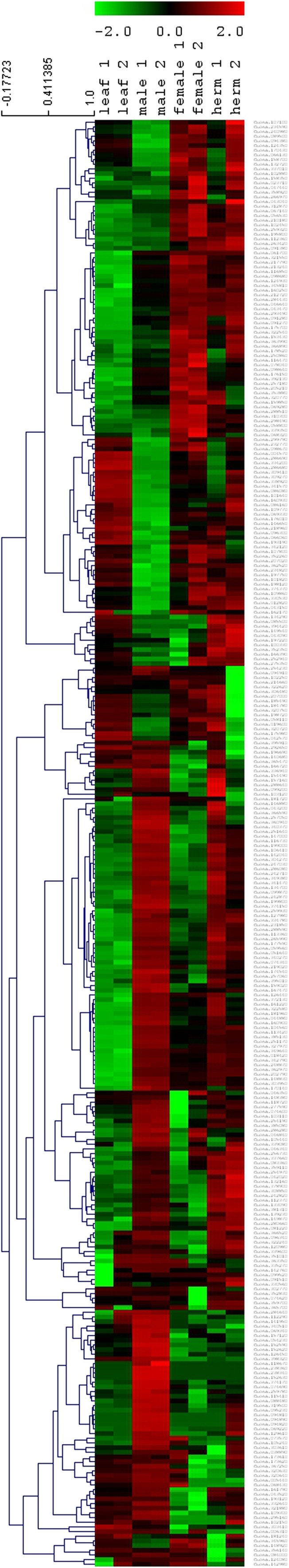
Heat map of differentially expressed genes in the leaf and shot apex tissues of male, female, and hermaphroditic lines. Gene expression levels are indicated at the top of the heat map, with red and green indicating upregulated and downregulated expression, respectively

Further, we analyzed DEGs with higher expression in the shoot apex based on the following three comparisons of flower sex types: female *vs* male (FvM), female *vs* hermaphrodite (FvH), and male *vs* hermaphrodite (MvH). We detected 310 DEGs as follows. A total of 260 DEGs were observed in the FvM comparison. Specifically, 111 genes were more highly expressed in female parts, including four genes that were not expressed in male parts. In contrast, 149 genes were more highly expressed in male parts, including 59 genes that were not expressed in female parts. The FvH comparison revealed 36 DEGs, three of which were more expressed in female parts and one gene that was not expressed in the hermaphrodite shoot apex. Additionally, 32 genes were more highly expressed in hermaphrodite parts, including 20 genes that were expressed exclusively in hermaphrodite samples. The MvH comparison detected 55 DEGs. Higher expression in male have shown 23 genes, 14 of which were only expressed in the male parts. Moreover, 31 genes were more highly expressed in hermaphrodite parts, three of which were expressed exclusively in hermaphrodite shoots. Quantity distributions of the 310 DEGs are presented in Fig. 2, while expression profiles are provided in Fig. 3 (Table S3).

### Confirmation of Illumina RNA-seq expression patterns by qPCR

**Fig. 4:**
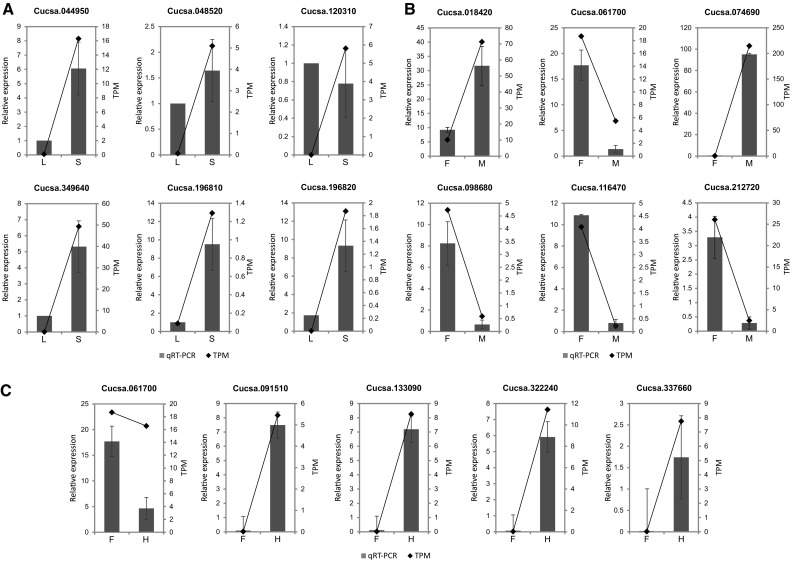
Validation of DEG analyses by quantitative PCR [qPCR: relative expression; RNA-seq: transcripts per million (TPM)]. **a** Genes differentially expressed between vegetative (L, leaves) and generative (S, shoots) tissues. **b** Genes differentially expressed between female (F) and male (M) shoots. **c** Genes differentially expressed between female (F) and hermaphroditic (H) shoots

To validate the analysis of DEGs based on RNA-seq data, 16 randomly chosen genes were analyzed in a qPCR assay. The qPCR and RNA-seq data for these genes were compared (Fig. 4, Table S4). Highly reliable reference genes (*CsCACS* and *CsTIP41*) were used to normalize the qPCR data according to the 2^−ΔΔCt^ method. The qPCR and transcriptome analyses produced consistent expression profiles for 15 of the 16 analyzed genes (*Cucsa.018,420*, *Cucsa.044950*, *Cucsa.048520*, *Cucsa.061700*, *Cucsa.074690*, *Cucsa.091510*, *Cucsa.098680*, *Cucsa.116470*, *Cucsa.133090*, *Cucsa.196810*, *Cucsa.196820*, *Cucsa.322240*, *Cucsa.212720*, *Cucsa.322240*, and *Cucsa.337660*). The exception was *Cucsa.120310*. This lack of consistency may have occurred at least partly because of differences in the sensitivity of the techniques and algorithms. However, 94% of the analyzed DEG profiles were positively validated by qPCR.

### Functional annotation

**Fig. 5:**
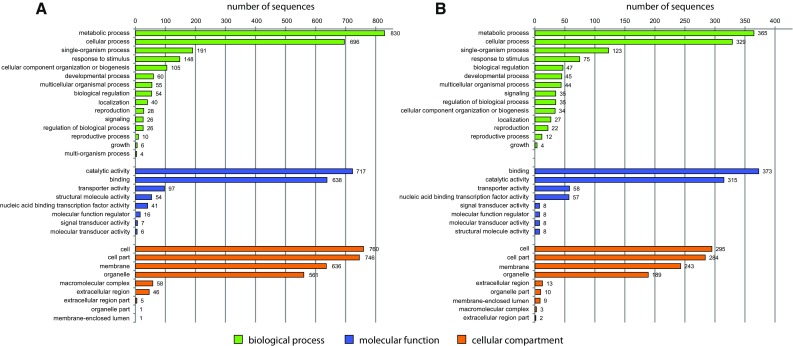
Histogram of GO analysis of DEGs detected in the comparison between leaves and the shoot apex. **a** Genes exhibiting upregulated expression in leaves (1919). **b** Genes exhibiting upregulated expression in the shoot apex (933)

Selected genes were annotated with GO terms. Genes that exhibited upregulated expression in the shoot apex and leaves were assigned to functional categories using Blast2GO software. These genes were further classified using a set of plant-specific GO slims, and clustered into three main groups (biological process, molecular function, and cellular component). Details regarding the GO analysis of DEGs are provided in Fig. 5. Within the biological process category, metabolic process, cellular process, and single-organism process were among the most highly represented groups for the genes with upregulated expression in the shoot apex and leaves. Within the molecular function category, catalytic activity and binding were the most represented groups (Table S5).

### Functional annotation of sex-specific DEGs

**Fig. 6:**
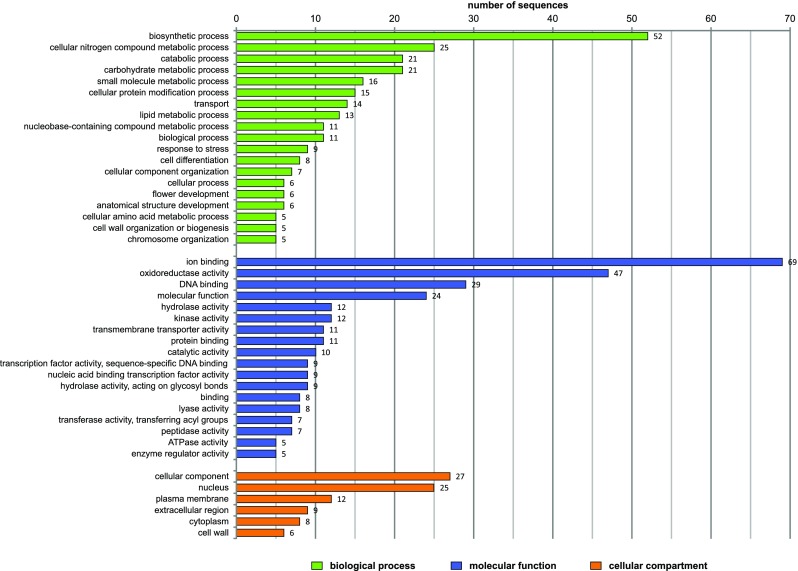
Histogram of GO analysis of 310 sex-specific DEGs. The GO terms assigned to more than five genes are presented

The 310 unigenes identified as sex-specific DEGs were annotated according to GO categories using the plant-specific GO slim. These DEGs were associated with 111 significantly enriched GO terms clustered into three main categories (Fig. 6). The most abundant GO terms in the biological process category were biosynthetic process, cellular nitrogen compound metabolic process, catabolic process, and carbohydrate metabolic process. In the molecular function category, ion binding and oxidoreductase activity were the most common GO terms. Meanwhile, in the cellular component category, cellular component and nucleus were the most abundant GO terms. Moreover, additional enriched GO terms were related to other processes and functions associated with flower development, including cell differentiation, flower development, and anatomical structure development (Table S6).

**Fig. 7:**
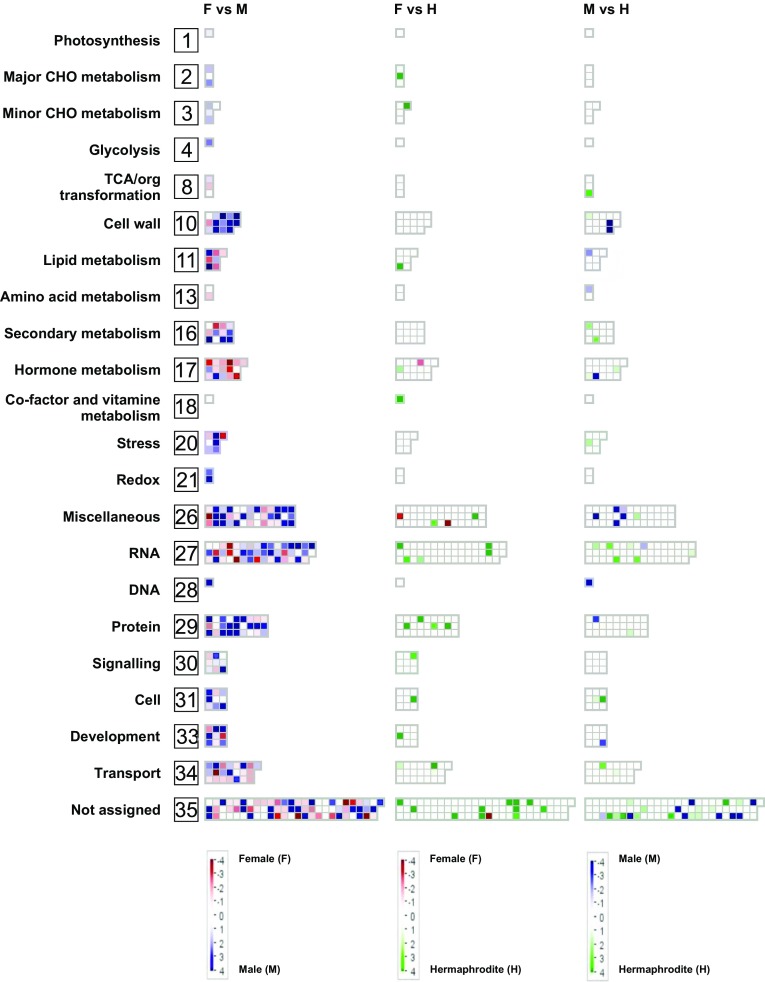
MapMan analysis of 310 DEGs based on the following three comparisons: female *vs* male, female *vs* hermaphrodite, and male *vs* hermaphrodite. Genes that were not differentially expressed in a specific comparison are indicated in white, genes upregulated in female are indicated in red, male are indicated in blue, and hermaphrodite are indicated in green

To visualize the dataset and further examine the genes related to sex determination, we completed a MapMan analysis, which determined the location and implied function for most of the 310 sex-specific DEGs. These genes were assigned to 22 out of 35 groups (Fig. 7, Table S7). The largest group included 47 genes, and was associated with RNA and the regulation of transcript levels (e.g., MADS box and homeobox). Another group (i.e., miscellaneous group) comprised 39 genes encoding various enzymes, such as oxidases, nitrilases, cytochrome P450, and peroxidases. A third group contained 27 genes encoding factors related to degradation, synthesis, post-translational modifications, and protein interaction pathways. Other groups were associated with hormone metabolism, signaling, transport, secondary metabolism, or the cell wall. In male shoots, the genes with upregulated expression levels were associated with miscellaneous, protein, or cell wall groups. Meanwhile, in female shoots, the genes with upregulated expression levels were responsible for hormone metabolism, lipid metabolism, or transport. The groups with the fewest genes were related to photosynthesis, glycolysis, vitamin cofactor metabolism, DNA synthesis, and chromatin structure (Fig. 7).

### Bioinformatic modeling of interactions among proteins encoded by DEGs

**Fig. 8:**
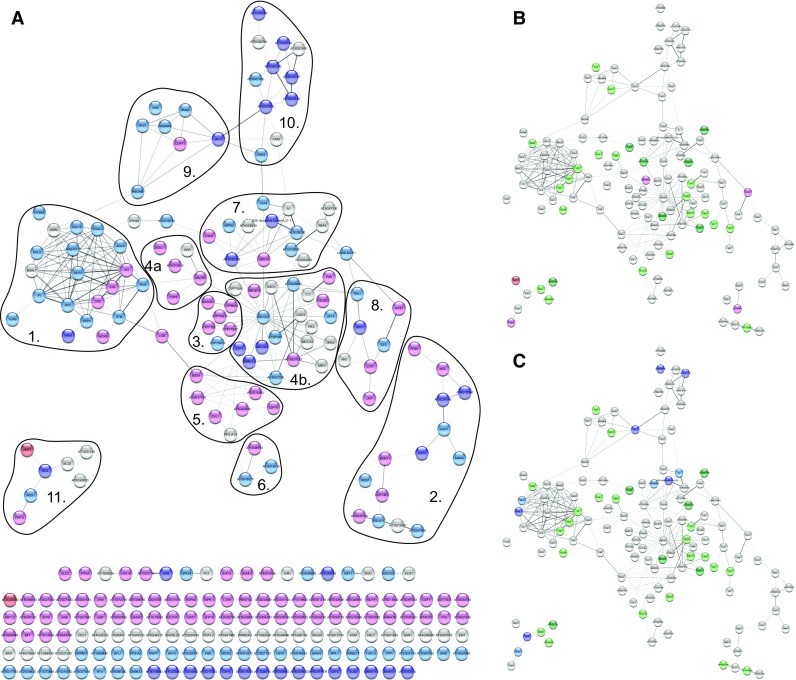
The results of in silico study of STRING software for protein–protein interaction network for 310 sex-specific DEGs. There were gene expression differences between the female and male lines (**a**). Proteins in red were detected only in female lines, while proteins in light red were more abundant in the female shoot apex than in the male shoot apex. Similarly, proteins in blue were present only in male lines, while proteins in light blue were more abundant in the male shoot apex than in the female shoot apex. Nodes in gray were not differentially expressed between the male and female lines. The thickness of edges corresponds to the confidence of the protein connection (thicker line = greater confidence in the connection). **b** Main network in the female *vs* hermaphrodite comparison; Proteins in red were detected only in female lines, while proteins in light red were more abundant in the female shoot apex than in the hermaphroditic shoot apex. Similarly, proteins in green were present only in hermaphrodite lines, while proteins in light green were more abundant in the hermaphrodite shoot apex than in the female shoot apex. Nodes in gray were not differentially expressed between the hermaphrodite and female lines. **c** Main network in the male *vs* hermaphrodite comparison. Proteins in blue were detected only in male lines, while proteins in light blue were more abundant in the male shoot apex than in the hermaphroditic shoot apex. Similarly, proteins in green were present only in hermaphrodite lines, while proteins in light green were more abundant in the hermaphrodite shoot apex than in the male shoot apex. Nodes in gray were not differentially expressed between the hermaphrodite and male lines. Networks were prepared using the STRING program and then visualized with Cytoscape

For the first time a molecular network of proteins connected to sex determination in cucumber was constructed based on the results of a STRING analysis (Fig. 8, Table S8). The network consisted of 269 nodes (each representing a protein) and 377 edges, with an average node degree of 2.79. Additionally, the average local clustering coefficient was 0.402 and the p-value of the protein–protein interaction was < 1.0e−16. These results imply the network content was based on non-random proteins. A total of 127 nodes were detected as a single component, while 18 nodes formed small two-component connections with mutual interactions. Additionally, 117 nodes were included in the main molecular network, and seven nodes formed a smaller network. Protein–protein interactions were significantly enriched, indicating that the detected proteins in the STRING database interact more with each other than would be expected for a random set of similar-sized proteins encoded in a genome. This enrichment implies that the proteins are at least partially biologically related in a group. In this study, we distinguished the following 11 groups in the main molecular network (Fig. 8): (1) transcription factors that mediate the transition from an inflorescence meristem to a floral meristem, floral meristem development, and regulation of floral organ identity; (2) enzymes (transferase, peptidase, esterase, and protease); (3) cytochromes; (4a) and (4b) represent the central nodes with ATP-binding transporters that use the energy from the binding and hydrolysis of ATP to transport various substrates across cellular membranes, thereby regulating many processes; (5) proteins associated with brassinosteroids and the regulation of development via ubiquitination-related molecules; (6) lipid metabolism; (7) proteins related to ubiquitination processes; (8) proteins from the ethylene and jasmonic acid signaling pathways; (9) metal ion binding; (10) pectin catabolic processes related to pollen maturation and the flowering stage; and (11) proteins related to ion transport, sugar metabolism, and programmed cell death (PCD).

**Fig. 9:**
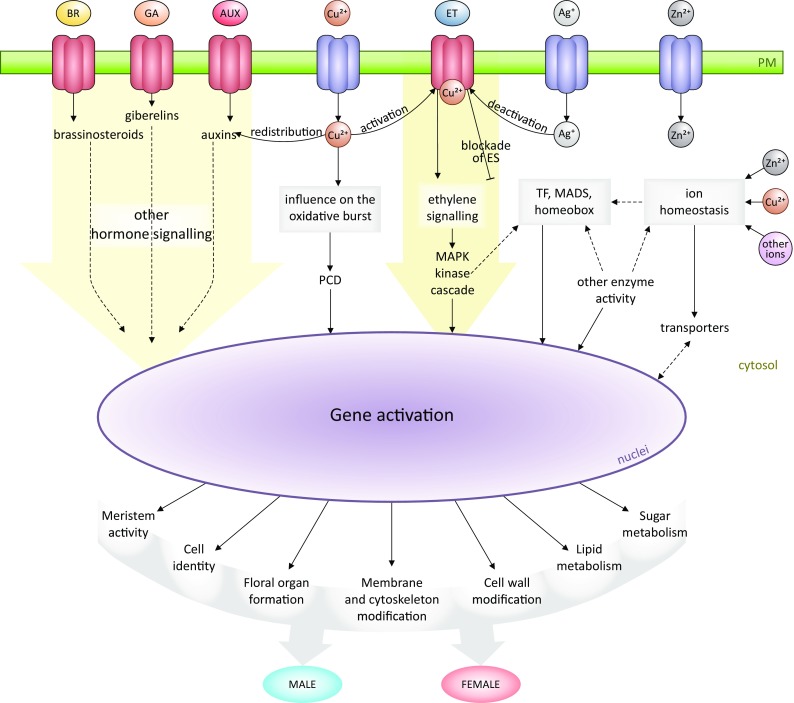
Proposed hypothetical model of the regulatory networks associated with sex determination in cucumber

The molecular network described herein may clarify the possible protein interactions mediating processes influencing sex determination in cucumber. To date, protein–protein interactions occurring during flower development have not been established because of the complexity of the relevant processes. Thus, an important part of flower morphogenesis studies involves elucidating the complex processes that occur during the formation of the floral meristem and organ primordia. The first event during floral development is the initiation of apical meristem cell differentiation, resulting in the production of a floral meristem. At the moment of differentiation, it is critical that the correct organs are created at the required locations and the duration of cell proliferation is appropriate. Furthermore, specific cells form the floral organ primordia, after which the flower bud starts to develop. Typically, the most intensive growth takes place in the lateral cells, from which sepals develop in the first whorl. Cells then differentiate into the petals of the second whorl. The stamen and pistil primordia appear in the third and fourth whorls, and grow very slowly. The primordia of generative organs in the third and fourth whorls have a different fate. Because each organ occupies a distinct whorl, cells in the shoot apex must assume the correct organization and position. It remains unclear which processes and mechanisms define the cell fate and determine the flower organ identity and sex (Kim et al. 2000). However, a very complex mechanism involving many genes is almost certainly required. To identify appropriate links between sex determination and flower morphogenesis in cucumber, we manually curated the in silico functional analyses (Table S9). We revealed a novel regulatory mechanism that provides new insights into flower development (Fig. 9).

#### Transcription factors

Transcription factors (TF) are important regulators of cellular processes in plant development. One group of TF, those involved in floral organ identity determination, is plant MADS-box. Analysis of homeotic floral mutants resulted in the formulation of a genetic model, named the ABC model (Coen and Meyerowitz 1991) and later was expanded to include classes D and E. This model, that explains how the combined functions of classes of genes determine the identity of the four flower organs. The development of sex-specific organs relies on the combinatorial and differential expression of such homeotic genes over time and space (Guo et al. 2015). Based on the quartet model, the A- and E-class complex [APETALA1 (AP1)– SEPALLATA (SEP)] specify the sepal identity; A-, B- and E-class proteins [AP1–SEP–AP3–PISTILLATA (PI)] specify petals; the B-, C- and E-class complex [AGAMOUS (AG)–SEP–AP3–PI] specify stamens; the C- and E-class complex (AG–SEP) specify carpels; and the D- and E-class complex [SEEDSTICK (STK)–SEP] specify ovules (Bowman et al. 2012; Guo et al. 2015). In this study, we identified genes related to transcription factors (10%) (Fig. 8; Tables 3, S3, and S9), including *AGAMOUS* (*Cucsa.251170*, *Cucsa.202790*, *Cucsa.362970*, *Cucsa.174540*, *Cucsa.248870*, and *Cucsa.212720*), *SEPALLATA* (*Cucsa.018420*, *Cucsa.327970*, and *Cucsa.349640*), *SEEDSTICK* (*Cucsa.061700*), *PISTILLATA* (*Cucsa.018420*), and *APETALA 3* (*Cucsa.342790*), which are expressed according to the MADS-box rules.

**Table 3:** Transcription factors encoded by the DEGs

Comparison	Gene ID	Description	Log2FC	Adj *p* value
Female FvM	*Cucsa.366890*	Basic helix–loop–helix (bHLH) DNA-binding	− 2.85	3,95E−02
Female FvM	*Cucsa.284430*	Plant-specific transcription factor YABBY	− 3.57	2,11E−36
Female FvM	*Cucsa.160250*	Basic helix–loop–helix (bHLH) DNA-binding	− 1.14	2.42E−03
Female FvM	*Cucsa.240980*	Basic-leucine zipper (bZIP) TF	− 1.02	8.97E−03
Female FvM	*Cucsa.175980*	PAR1 protein	− 1.56	1.78E−02
Female FvM	*Cucsa.160930*	transcription factor-related	− 1.14	2.65E−02
Male FvM	*Cucsa.352830*	PHD finger transcription factor, putative	6.58	2.04E−05
Male FvM	*Cucsa.126450*	NAC domain-containing protein 25	2.54	9.37E−06
Male FvM	*Cucsa.074600*	NAC domain-containing protein 2	2.87	7.59E−05
Male FvM	*Cucsa.366520*	AP2/B3-like transcriptional factor	6.70	1.85E−10
Male FvM	*Cucsa.366590*	AP2/B3-like transcriptional factor	1.54	1.80E−02
Male FvM	*Cucsa.265990*	basic helix–loop–helix (bHLH) DNA-binding	1.96	2.34E−03
Male FvM	*Cucsa.199800*	bZIP transcription factor family protein	2.49	1.42E−13
Male FvM	*Cucsa.242870*	bZIP transcription factor family protein	3.18	2.97E−08
Male FvM	*Cucsa.385130*	bZIP transcription factor family protein	1.50	5.10E−03
Male FvM	*Cucsa.288590*	myb domain protein 68	3.84	2.18E−15

We also detected TF like *HAT5* (*Cucsa.159020*), which reportedly encodes an essential protein for the proper development of basal and lateral organ boundaries (Rutjens et al. 2009), and two genes with a *WUSCHEL* (*WUS*) domain female-specific homeobox-3 (*HB*-*3*)/*WOX 9* (*Cucsa.046640*) and male-specific *WUS* (*Cucsa.043520*). The WUSCHEL promotes stem cell proliferation. Based on literature reports, WUS and AG interact with each other. The initial expression of *AG* is activated by the WUS, but the accumulation of AG and also involvement of other proteins leads to *WUS* repression (Liu et al. 2011) what influence on terminating floral stem activities (Guo et al. 2015).

*KNOX STM SHOOT MERISTEMLESS* (*Cucsa.148830*) encodes a protein belonging to the KNOTTED1-like TALE (KNAT) class I family of transcription factors and could influence meristem activity. The KN1-related homeobox KNOX proteins may suppress cell differentiation (Kerstetter et al. 1997) and also repress gibberellic acid (GA) biosynthesis, which in turn may induce cell differentiation (Hay et al. 2002). The LOB protein (*Cucsa.098680*), which appears to be a female-specific protein, contributes to pollen development, as reported by Xu (Xu et al. 2016). Moreover, in other study, it was proven that LOB interacts with KNOX proteins to maintain stem cell identity (Rast and Simon 2012) and also negatively regulates brassinosteroid accumulation (Bell et al. 2012).

We identified other transcription factors (5%) that regulate various developmental processes, but any relationships with sex determination in cucumber have not been established till now. Some of these transcription factors (e.g., belonging to the large NAC domain family containing protein or bHLH protein family) were also detected in an earlier study devoted to sex expression in gynoecious and hermaphroditic cucumber lines (Guo et al. 2010). Transcription factors with typical binding motifs, such as bZIP, MYB, and a zinc finger, are inducible by many signals (Ambawat et al. 2013).

The factors responsible for floral organ identity (e.g., homeobox) and other transcription factors control the expression of many genes involved in diverse metabolic processes, including responses to different hormones such as ethylene (Han et al. 2016), cytokinins, auxins, gibberellins, and jasmonic acid (Thomson et al. 2017). These hormones also mediate various activities during floral bud development, starting from meristematic activity, as well as organ pattern formation and maturation (Chandler 2011).

#### Hormones

We identified genes (6.7%) correlated with the transduction or synthesis of hormones like auxins, cytokinins, ethylene (promotes femaleness), gibberellins (promotes maleness), abscisic acid (ABA), and brassinosteroids (Tables 4, S3, S9).

**Table 4:** Differentially expressed genes associated with hormone signaling

Comparison	Gene ID	Description	Hormone	Log2FC	Adj *p* value
Female FvM	*Cucsa.069330*	Protein phosphatase 2C	ABA	− 1.60	4.88E−03
Female FvM	*Cucsa.043470*	SAUR-like auxin-responsive	Auxin	− 1.33	1.03E−04
Female FvM	*Cucsa.178520*	Auxin efflux	Auxin	− 3.12	9.86E−03
Female FvM	*Cucsa.320720*	Auxin-Induced	Auxin	− 1.45	3.52E−03
Female FvM	*Cucsa.310300*	Indoleacetic acid-induced	Auxin	− 1.67	7.42E−08
Female FvM	*Cucsa.232770*	BRI1 kinase inhibitor 1	Brassinosteroids	− 1.28	2.66E−02
Female FvM	*Cucsa.299790*	Cytokinin oxidase 7	Cytokines	− 1.21	8.98E−03
Female FvM	*Cucsa.234590*	S-adenosyl-l-methionine methyltransferases	Ethylene	− 3.33	2.75E−02
Female FvM	*Cucsa.298490*	ethylene-dependent gravitropism-deficient	Ethylene	− 1.15	4.02E−03
Female FvM	*Cucsa.069280*	Ethylene-responsive element binding	Ethylene	− 1.09	1.79E−03
Female FvM	*Cucsa.322540*	Methionine gamma-lyase	Ethylene	− 1.47	3.28E−06
Female FvM	*Cucsa.288510*	1-Aminocyclopropane-1-carboxylate synthase 7	Ethylene	− 5.04	1.03E−12
Female FvM	*Cucsa.086080*	Gibberellin 2-oxidase 8	Gibberellin	− 1.73	9.03E−03
Female FvM	*Cucsa.004570*	Gibberellin 3-oxidase 1	Gibberellin	− 1.63	3.64E−03
Female FvM	*Cucsa.159850*	GAST1 protein homolog 3	Gibberellin	− 3.11	1.39E−03
Herma FvH	*Cucsa.359110*	Pyridoxine biosynthesis	Auxin	8.17	1.73E−12
Herma FvH	*Cucsa.132160*	Tetratricopeptide repeat (TPR)-like	Ethylene	3.64	2.65E−02
Herma MvH	*Cucsa.330530*	Response regulator 9	Gibberellin	2.07	2.22E−03
Male FvM	*Cucsa.330560*	TRAF-like family protein	ABA	Inf	4.66E−03
Male FvM	*Cucsa.059560*	Auxin-responsive GH3	Auxin	2.28	5.89E−05
Male FvM	*Cucsa.044880*	Ethylene-forming enzyme	Ethylene	1.73	3.86E−07

Ethylene is important for the development of female flowers. The mechanism by which ethylene triggers sex determination in cucumber has been extensively studied. The first step of the ethylene signaling cascade involves the perception of the hormone by a family of ethylene receptors (ETR1, ETR2, ERS1, ERS2, and EIN4) and then the signal processing by downstream proteins as follows: Ethylene receptors → CTR → EIN2 → EIN3/EIN3-like → ERF → response (Hubert et al. 2014). We identified several ethylene-related genes that were highly expressed in female lines, and were correlated with ethylene biosynthesis. These genes included those encoding methionine (*Cucsa.322540*), an S-adenosyl-l-methionine-dependent methyltransferase superfamily protein (*Cucsa.234590*), and 1-aminocyclopropane-1-carboxylate synthase 7 (*Cucsa.288510*). The expression of these genes was also correlated with the expression of genes mediating ethylene responses, such as the ethylene-dependent gravitropism-deficient protein (*Cucsa.298490*), ethylene-responsive element-binding protein (*Cucsa.069280*), and the tetratricopeptide repeat-like superfamily protein (*Cucsa.132160*). The only gene that was more highly expressed in male lines than in female lines was *Cucsa.044880*, which encodes an ethylene-forming enzyme, 1-aminocyclopropane-1-carboxylate oxidase 4. Interestingly, two genes correlated with the biosynthesis of methionine, which is the main amino acid used for ethylene synthesis, encode MTO1 pyridoxal phosphate-dependent transferases (Hacham et al. 2013). The expression of these two genes was differentially regulated in male (*Cucsa.166720*) and female (*Cucsa.392130*) cucumber lines. The high expression levels for the ethylene-related genes in the female lines are consistent with the findings of a previous study on sex determination in cucumber that proved that the ethylene contents and *ACS* expression levels are higher in female lines than in male or hermaphroditic lines (Boualem et al. 2008, 2015; Li et al. 2009; Mibus and Tatlioglu 2004; Trebitsh et al. 1997).

Auxin effects induce femaleness by increasing the ethylene level (Yin and Quinn 1995). In this study, with the exception of one male-specific gene (*Cucsa.059560*), all of the identified genes were highly expressed in female lines and encode proteins that induce or transduce auxin signals. These observations are consistent with published data. Probably, female plants produce relatively large amounts of auxin and transducers, which are needed to enhance the production of female flowers. The cytokinin oxidase *CKX7* gene (*Cucsa.299790*) is also highly expressed in females. How these hormones influence sex determination in plants has not been determined, but will be investigated in our future studies.

Gibberellins also affect sex differentiation in cucumber, but unlike auxin and ethylene, they influence the formation of male flowers. Treating cucumber plants with exogenous GA inhibits ethylene biosynthesis by suppressing *CsACS1G* expression. In females, gibberellin-related genes are highly expressed. These genes include those encoding oxidases (*Cucsa.004570* and *Cucsa.086080*), response regulators (*Cucsa.330530*), and the gibberellic acid stimulated-like protein (*Cucsa.159850*). The involvement of GA in cucumber flower formation was also described by Zhang et al. (2014). They identified *CsGAMYB1* as a positive regulator of the GA signaling pathway in cucumber, which presumably promotes male flower development and inhibits ethylene production and the formation of female flowers.

There is no clear connection between flower formation and ABA or brassinosteroids. The genes encoding ABA-related protein phosphatase 2C were revealed to be differentially expressed (Pawełkowicz et al. 2016b). These observations were confirmed by our RNA-seq data. Additionally, the female-specific protein phosphatase 2C (*Cucsa.069330*) is a negative regulator of ABA and the tumor necrosis factor receptor-associated factor (*Cucsa.330560*), which is a positive component of the ABA signaling pathway (Bao et al. 2014). Furthermore, a brassinosteroid-correlated gene (*Cucsa.232770*) encoding BRI1 kinase inhibitor 1 was highly expressed in females. The above mentioned genes may modulate signal transduction pathways and change the sensitivity of female tissues to certain hormones. Plant hormones and sex determination are not highly correlated, but the crosstalk involving a hormone-signaling cascade may affect cucumber flower formation. Ethylene eliminates the effects of the other hormones and is a major sex-regulating factor.

#### Metal ions

The relationship between metal ion homeostasis and ethylene signaling has been revealed (Hirayama and Alonso 2000). In this study, we determined that 8.7% of the DEGs (Tables 5, S3, S9) are correlated with metal ion processes, especially those involving copper (3.2%) (Fig. 8 bin 9) and zinc (2.9%).

**Table 5:** Differentially expressed genes related to hormone signaling

Comaprison	Gene ID	Description	Specifity	Log2FC	Adj *p* value
Female FvM	*Cucsa.146650*	Copper transporter 1	Copper	− 2.31	4.75E−03
Female FvM	*Cucsa.185490*	Copper amine oxidase	Copper	− 1.74	2.16E−06
Female FvM	*Cucsa.176010*	SOUL heme-binding	Ion binding	− 1.43	3.64E−03
Female FvM	*Cucsa.162170*	Ammonium transporter 2	Ion binding	− 4.81	4.01E−02
Female FvM	*Cucsa.312870*	Transcription factor jumonji	Zinc	− 1.03	9.86E−03
Female FvM	*Cucsa.116850*	CCCH-type zinc finger	Zinc	− 0.87	4.48E−02
Female FvM	*Cucsa.078040*	Zinc-binding dehydrogenase	Zinc	− 0.98	1.46E−02
Female FvM	*Cucsa.205210*	Zinc finger protein 6	Zinc	− 1.38	2.24E−04
Female FvM	*Cucsa.175700*	Zinc finger protein 10	Zinc	− 3.39	4.31E−09
Herma FvH	*Cucsa.112370*	Uclacyanin 1	Copper	4.63	8.54E−05
Herma FvH	*Cucsa.337660*	Werner syndrome-like exonuclease	Ion binding	9.72	8.69E−22
Herma FvH	*Cucsa.322240*	RING/FYVE/PHD zinc finger	Zinc	10.35	1.57E−09
Herma FvH	*Cucsa.280660*	Zinc finger C-x8-C-x5-C-x3-H type	Zinc	2.27	5.26E−03
Herma MvH	*Cucsa.109860*	4-Hydroxy-3-methylbut-2-enyl diphosphate reductase	Ion binding	2.27	5.76E−03
Male FvM	*Cucsa.152590*	SKU5 similar 12	Copper	7.69	1.47E−03
Male FvM	*Cucsa.105440*	Blue-copper-binding protein	Copper	8.12	2.37E−03
Male FvM	*Cucsa.177590*	NRAMP metal ion transporter 6	Copper	2.08	1.47E−03
Male FvM	*Cucsa.112290*	SKU5 similar 12	Copper	5.24	4.07E−02
Male FvM	*Cucsa.152620*	SKU5 similar 12	Copper	6.65	1.46E−02
Male FvM	*Cucsa.152630*	SKU5 similar 12	Copper	Inf	1.39E−03
Male FvM	*Cucsa.251640*	SKU5 similar 1	Copper	1.77	4.03E−05
Male FvM	*Cucsa.170140*	SNF1-related protein kinase 2.5	Ion binding	Inf	2.60E−02
Male FvM	*Cucsa.319500*	subtilase family protein	Ion binding	4.86	4.23E−02
Male FvM	*Cucsa.398320*	2-Oxoglutarate Fe(II)-dependent oxygenase	Ion binding	5.71	4.79E−09
Male FvM	*Cucsa.367250*	Heavy metal atpase 5	Ion binding	1.59	4.23E−02
Male FvM	*Cucsa.173610*	BED zinc finger	Zinc	Inf	3.63E−03
Male FvM	*Cucsa.259930*	Zinc ion binding; DNA binding	Zinc	3.65	4.02E−09

There is biochemical evidence indicating that copper is an important for plant growth due to its contribution in many processes including oxidative stress responses, cell wall metabolism (Yruela 2005), auxin redistribution (Yuan et al. 2013), and ethylene perception and signaling (Hirayama and Alonso 2000). Copper ions induce the ethylene biosynthesis pathway, by upregulating the expression of *ACS* genes (Mao et al. 2015; Wang et al. 2002; Yang and Hoffman 1984; Zhang et al. 2017), which are referred to as sex genes in cucumber. A previous investigation confirmed that a CuSO_4_ treatment promotes the accumulation of reactive oxygen species and callose deposition in the cell wall, while also activating MAP kinase signaling (Liu et al. 2015). Several genes (*Cucsa.112290*, *Cucsa.152590*, *Cucsa.152620*, *Cucsa.152630*, and *Cucsa.251640*) that were highly expressed in male lines encode glycoproteins that are structurally related to multiple copper oxidases (e.g., SKU5). Previous studies by Loraines’s group revealed that *SKU* is highly expressed in mature pollen grains, with the encoded protein localized in the plasma membrane and cell wall (Loraine et al. 2013; Wang et al. 2008). Our results suggest that SKU5 may have a regulatory function during cucumber flower development via its effects on cell walls as well as during pollen formation. Other DEGs related to copper, encoded blue copper proteins (BCPs) (Table 5). According to the literature, these proteins may participate in electron transfers, affect redox reactions, and influence cell wall formation in plants (Nersissian et al. 1998), while functioning with other molecules (e.g., cytochromes) (Printz et al. 2016). In a recent study, type 1 BCPs were observed to be secreted from the pistil to guide the pollen tube and grain toward the pistil (Rhee et al. 2015). These BCPs are reportedly male sterility factors in some cotton lines (Suzuki et al. 2013).

Little is known about copper uptake and transport in plants. In this study, we identified genes encoding possible copper transporters (*Cucsa.146650*, *Cucsa.177590*, *Cucsa.367250*, and *Cucsa.177590*). Copper influences ethylene signaling because ethylene receptors are copper dependent (Alonso et al. 1999; Hirayama and Alonso 2000; Rodriguez et al. 1999). Ethylene, which binds to receptor, requires copper as a cofactor. Interestingly, ethylene receptor activities may be affected by phosphorylation. The phosphorylation of ETR1 requires an ethylene-binding domain and copper (Bisson and Groth 2012). Decreases in phosphorylation may be blocked by the presence of silver ions. An AgNO_3_ treatment of cucumber plants reportedly can influence flower sex determination by inducing the formation of male flowers (Malepszy and Niemirowicz-Szczytt 1991). Thus, we propose that this is correlated with a decrease in the sensitivity of ethylene receptors. Nevertheless, it appears that copper homeostasis is very important for ethylene perception and signaling.

The gene encoding COPT1, which belongs to another copper transporter family, is highly expressed in females. This protein enables copper in the soil to enter plant cells (Sancenón et al. 2003). The uptake of exogenous copper essentially depends on COPT1 in the plasma membrane. The entry of large amounts of Cu(I) generates a burst of OH^−^, which activates Ca^2+^ influx channels and induces the opening of K^2+^ efflux channels, thereby activating a caspase-like protein-induced PCD (Rodrigo-Moreno et al. 2013). These changes may be responsible for inhibiting organ growth (e.g., root elongation) in *A. thaliana* (Rodrigo-Moreno et al. 2013). A previous study revealed some PCD occurring in cucumber male flower buds, which may be evidence of stamen inhibition through PCD (Delorme et al. 2000). However, it is still unclear whether such PCD is correlated with copper and how this phenomenon may be related to sex determination in cucumber flowers.

In summary, we identified proteins correlated with copper and its transporters, and determined that the corresponding genes are differentially expressed between sex types. We assume that the expression-level differences result in diverse copper transport in cells. This ultimately leads to differences in the quality and quantity of the copper available to ethylene receptors, which may affect receptor activity. Receptor efficiency is the basis for the signal transmission in the ethylene signaling pathway, or the redistribution of auxin. Finally, changes in copper homeostasis may induce PCD in unwanted floral bud organs, leading to the formation of unisexual flowers. Additionally, diversity in the expression of genes encoding proteins correlated with copper and homeostasis in developing floral buds as well as the co-participation of ions in specific processes affecting flower morphogenesis may influence the cucumber sex determination.

Another group of DEGs was related to zinc. Like copper, zinc can influence ethylene production (Mertens et al. 1999). We identified several genes encoding zinc finger proteins specific to males (*Cucsa.173610* and *Cucsa.259930*), females (*Cucsa.116850*, *Cucsa.078040*, *Cucsa.205210*, and *Cucsa.175700*), and hermaphrodites (*Cucsa.322240* and *Cucsa.280660*). Zinc finger (Znf C2H2) domains are relatively small protein motifs that contain multiple finger-like protrusions that make tandem contacts with their target molecule. These domains bind zinc or other metals (Gamsjaeger et al. 2007). In a recent study of banana fruit ripening, ethylene was observed to activate C2H2 proteins, which repressed the expression of ethylene biosynthetic genes (*ACS1* and *ACO1*) by directly binding to the promoters (Han et al. 2016). On the basis of a study examining *Cucumis melo*, the *gy* sex gene is presumably a member of the *WIP* family. The *WIP* gene in melon encodes a transcription factor domain C2H2 zinc finger protein (Boualem et al. 2015, 2016; Martin et al. 2009; Renner 2016). This gene controls the development of male flowers, as the loss-of-function mutant carrying the recessive form produces only female flowers (Boualem et al. 2008). Little is known about the cucumber *WIP* gene family and its correlation with sex determination. A recent study investigating ethylene perception and the subsequent signal transmission revealed that cucumber CsWIP1 binds to the *CsACO2* promoter to repress expression. The *CsACO2* gene is necessary for the production of female flowers, and the encoded protein functions co-operatively with CsACS11 to provide the necessary ethylene for carpel development (Chen et al. 2016). It appears likely that zinc finger proteins are associated with sex determination in cucumber, although this possibility will need to be experimentally verified.

#### Cell wall and cytoskeleton

Proper cell wall assembly is very important, especially during pollen formation and pollen tube growth. The improper accumulation of callose and pectins in the cell wall results in abnormal pollen tube growth (Sede et al. 2018). Pectins are an important component of the pollen tube cell wall. Consequently, pectin-modifying proteins are important for rearranging the cell wall. Thus, pectin clearly affects the structure and chemical composition of pollen tube cell walls (Mollet et al. 2013). Moreover, pectin was observed between microspores, which is very important for the creation of the microspore tetrad. In mutants in which many of the pectin esterase-associated genes were not expressed, the resulting microspore tetrads dissolved (Jakobsen et al. 2005). In this study, we confirmed that genes encoding proteins associated with the production and modification of pectins and cell wall rearrangements are in one related group. Additionally, these genes were more highly expressed in male flowers than in female or hermaphroditic flowers (Fig. 8 bin 10, Tables 6, S3, and S9). Proper pollen development is among other factors, one of the basis of successful fertilization and reproduction. We identified several genes related to pectin metabolism in the male cucumber lines, including pectin invertase or methylesterase (*Cucsa.181720*, *Cucsa.374170*, *Cucsa.069220*, *Cucsa.069220*, *Cucsa.374150*, and *Cucsa.286280*). According to a previous study, manipulating pectin within the cell wall may also affect organ emergence in the shoot apex (Peaucelle et al. 2011). In *A. thaliana*, the gene encoding a pectin lyase-like (PLL) protein is highly expressed in the stamen because of the regulated stamen abscission during floral bud cell separations (Sun et al. 2010). In this study, we also identified genes encoding PLL proteins (*Cucsa.005440*, *Cucsa.339080*, *Cucsa.099870*, *Cucsa.124080*, and *Cucsa.142040*) that are likely involved floral bud cell separation. During periods of intensive growth, like in differentiating buds, dynamic remodeling of the cell wall occurs. We identified genes (10%) correlated with cell wall modifications as well as the cytoskeleton and membranes. These genes were differentially expressed among the floral bud sex types. In plants, organ emergence requires cell expansion, which is controlled by turgor pressure and cell wall relaxation. As such, organ emergence likely requires changes to the cell wall mechanics. The manipulation of pectins within the cell walls of the meristem affects organ emergence. These cell wall changes are accompanied by cytoskeleton modifications. We detected the profilin gene (*Cucsa.116730*) as a DEG that was highly expressed in the male cucumber line. The encoded actin-binding protein contributes to the dynamic turnover and restructuring of the cytoskeleton. Additionally, profilin is important for the spatial and temporal control of microfilament growth, which is an essential process for cellular locomotion and changes to cell shapes (Gunning et al. 2015). Among other genes, we identified the gene encoding the TUA4 tubulin (*Cucsa.219020*) required for transporting cell wall components (Holmes-Davis et al. 2005), the gene encoding formin (*Cucsa.103120*), which mediates the elongation of filaments (Gunning et al. 2015), and the gene encoding the LIM domain-containing protein (*Cucsa.101820*) responsible for cytoskeleton organization and protein interactions. During floral bud development, the cell wall, cytoskeleton, tubules, filaments, and plasma membrane are considerably modified. Cell differentiation alters cell plasticity via many morphological and biochemical modifications to the cell membrane networks.

**Table 6:** Differentially expressed genes related to the cell wall and membrane

Comparison	Gene ID	Description	specificity	Log2FC	Adj p-val
Female FvM	*Cucsa.309270*	Cellulose synthase-like B3	Cell wall	− 2.17	8.36E−03
Female FvM	*Cucsa.170430*	Glycine-rich protein	Cell wall	− 2.75	1.58E−22
Female FvM	*Cucsa.124350*	Plasma membrane intrinsic protein 2A	Membrane	− 2.33	1.09E−03
Female FvM	*Cucsa.345810*	ENTH/ANTH/VHS superfamily protein	Membrane	− 2.21	1.62E−03
Herma FvH	*Cucsa.083360*	Endomembrane protein 70 protein family	Membrane	Inf	2.20E−06
Herma FvH	*Cucsa.381310*	Rhomboid-related intramembrane serine protease	Membrane	2.86	2.16E−02
Herma MvH	*Cucsa.101820*	LIM domain-containing protein	Cytoskeleton	1.57	5.64E−04
Male FvM	*Cucsa.303610*	Cellulase (glycosyl hydrolase family 5) protein	Cell wall	Inf	5.54E−07
Male FvM	*Cucsa.322580*	Glycosyl hydrolase 9B13	cell wall	1.44	1.10E−04
Male FvM	*Cucsa.146880*	Glycosyl hydrolase family protein	Cell wall	1.36	3.22E−02
Male FvM	*Cucsa.292650*	Xyloglucan endotransglucosylase/hydrolase 32	Cell wall	2.29	2.16E−02
Male FvM	*Cucsa.181720*	Plant invertase/pectin methylesterase inhibitor	Cell wall	Inf	2.17E−04
Male FvM	*Cucsa.374170*	Plant invertase/pectin methylesterase inhibitor	Cell wall	5.85	3.17E−03
Male FvM	*Cucsa.099870*	Pectin lyase-like superfamily protein	Cell wall	2.12	1.10E−03
Male FvM	*Cucsa.124080*	Pectin lyase-like superfamily protein	Cell wall	Inf	1.46E−02
Male FvM	*Cucsa.142040*	Pectin lyase-like superfamily protein	Cell wall	2.23	6.20E−08
Male FvM	*Cucsa.069220*	Plant invertase/pectin methylesterase inhibitor	Cell wall	7.10	3.82E−03
Male FvM	*Cucsa.374150*	Plant invertase/pectin methylesterase inhibitor	Cell wall	8.76	7.71E−04
Male FvM	*Cucsa.286280*	Plant invertase/pectin methylesterase inhibitor	Cell wall	7.14	5.89E−05
Male FvM	*Cucsa.005440*	Pectate lyase family protein	Cell wall	Inf	8.32E−04
Male FvM	*Cucsa.339080*	Pectate lyase family protein	Cell wall	10.24	2.24E−06
Male FvM	*Cucsa.103120*	formin homolog 6	Cytoskeleton	1.25	1.21E−02
Male FvM	*Cucsa.231850*	thioredoxin H-type 1	Membrane	2.52	1.61E−05
Male FvM	*Cucsa.161790*	H(+)-ATPase 5	Membrane	2.17	1.51E−09
Male FvM	*Cucsa.134700*	Leucine-rich repeat transmembrane protein kinase	Membrane	2.88	1.15E−04
Male FvM	*Cucsa.116730*	profilin 4	Membrane	3.52	1.19E−05
Male FvM	*Cucsa.140680*	Leucine-rich repeat transmembrane protein kinase	Membrane	1.05	1.54E−02
Male FvM	*Cucsa.339600*	Nodulin MtN3 family protein	Membrane	Inf	6.81E−09
Male FvM	*Cucsa.142760*	Early nodulin-like protein 20	Membrane	Inf	2.24E−06
Male FvM	*Cucsa.157120*	Nodulin MtN3 family protein	Membrane	4.53	4.88E−09
Male FvM	*Cucsa.303950*	Nodulin MtN3 family protein	Membrane	1.18	2.97E−02

We also identified many membrane-related genes (Tables 6, S3, and S9) with specific expression patterns in different floral bud sex types. The encoded proteins are likely to function in the reconstruction and organization of developing sex organs. Several genes were identified as nodulin genes (*Cucsa.142760*, *Cucsa.339600*, *Cucsa.157120*, and *Cucsa.303950*), which are highly expressed in male flowers because of their specific role related to pollen formation. An earlier *A. thaliana* study indicated the nodulin protein with MtN3 domains is a plasma membrane protein specific to male flowers because it is required for the exine pattern of microspores (Guan et al. 2008).

Ethylene and auxins influence cell wall elongation, growth, and inhibition. Ethylene and auxin gradients between cells may affect several cellular activities, including cell extension, cell differentiation (Bashline et al. 2014; Osborne 1979), and remobilization of cell wall pectins (Zhu et al. 2016). It is likely that the changes occurring in the cell wall during the differentiation of shoot apical cells are part of the processes mediating sex determination in cucumber flowers because of their effects on the differentiation of generative organs.

#### Transporters and cytochromes

Signals received by cells affect cell activities. Many enzymes are involved in the transmission of signals, as are some transporters. The ABC transporter family is very large, with members mediating transmembrane transport and/or regulating other transporters (Rea 2007). The ABC proteins involve ATP during the transport of diverse substrates (e.g., lipids, heavy metal ions, sugars, amino acids, peptides, and secondary metabolites) (Jasinski et al. 2003) as well as auxin (Rea 2007) across various membranes. The ABC transporters encoded by the genes identified in this study (*Cucsa.139230*, *Cucsa.158700*, *Cucsa.074620*, and *Cucsa.012820*) react with many proteins that vary regarding molecular function (Fig. 8 bin 4a and b, Table S9). Interestingly, the expression patterns for these genes differed among the male, female, and hermaphroditic plants, suggesting the encoded proteins serve as transporters for different processes related to the determination of flower sex type. Specifically, ABCG31 is specific to male flowers, and is included in the central node of bin 4b. This protein was observed to interact with 23 proteins in main molecular network (Fig. 8), implying that it is a multitasking protein. Choi et al. proved that ABCG31 influences pollen coat maturation, possibly by regulating the distribution of steryl glycosides, thereby altering the membrane properties at the pollen surface, which affects pollen fitness (Choi et al. 2014). We identified several other transport-related genes (3.87%) (Tables 7, S3, and S9) which are involved in many processes in the differentiating cell, but their direct impact on sex expression is not clear.

**Table 7:** Differentially expressed genes related to transport

Comparison	Gene ID	Description	Specificity	Log2FC	Adj *p* value
Female FvM	*Cucsa.158700*	ABC transporter family protein	Transport	− 1.06	1.40E−02
Female FvM	*Cucsa.309410*	Major facilitator superfamily protein	Transport	− 0.98	1.45E−02
Female FvM	*Cucsa.339350*	Major facilitator superfamily protein	Transport	− 1.62	3.14E−03
Female FvM	*Cucsa.338920*	NOD26-like intrinsic protein 1;2	Transport	− 1.84	4.43E−05
Female FvM	*Cucsa.193190*	NOD26-like intrinsic protein 5;1	Transport	− 1.68	2.43E−05
Female FvM	*Cucsa.217790*	Sulfate transporter 1;1	Transport	− 1.46	1.47E−03
Herma MvH	*Cucsa.012820*	Multidrug resistance-associated protein 14	Transport	1.25	2.25E−02
Male FvM	*Cucsa.139230*	Pleiotropic drug resistance 3	Transport	2.91	3.31E−03
Male FvM	*Cucsa.074620*	ABC-2 type transporter family protein	Transport	8.28	2.90E−12
Male FvM	*Cucsa.142980*	Major facilitator superfamily protein	Transport	Inf	2.65E−02
Male FvM	*Cucsa.254190*	Major facilitator superfamily protein	Transport	1.84	1.63E−02
Male FvM	*Cucsa.160900*	MATE efflux family protein	Transport	1.53	8.18E−03

The ABCG31 transporter also interacts with cytochromes (Fig. 8 bins 4 b and 3). The DEGs included several genes encoding cytochromes (2.9%), such as *BCS1* (*Cucsa.043090*), *BAS1* (*Cucsa.293490*), *CYP75B1* (*Cucsa.149540*), *CYP71B2* (*Cucsa.286690*), *CYP71B37* (*Cucsa.286680*), *CYP716A1* (*Cucsa.356140*), *CYP72A15* (*Cucsa.058110*), and *CYP88A3* (*Cucsa.257180*). These genes were highly expressed in the female shoot apex. In contrast, only one cytochrome gene, *CYP78A10* (*Cucsa.113360*), was highly expressed in the male shoot apex (Tables S3 and S9). Cytochromes are involved in flower morphogenesis and reproduction (Larkin 1994; Petkova-Andonova et al. 2002). Cytochrome P450 regulates many important cell processes affecting plant growth and development. Some P450 genes are expressed during cell wall synthesis, and the encoded proteins are important for regulating plant hormone metabolism (Jun et al. 2015). Cytochrome P450 is involved in the biosynthesis of auxins, cytokinins, jasmonic acid, gibberellins, and ABA (Jun et al. 2015), and also affects ethylene metabolism (Chang et al. 2014). According to Chang et al. (2014), repression by the ethylene receptor ETR1 depends on an integral membrane protein, REVERSION-TO-ETHYLENE SENSITIVITY1 (RTE1), Cytochrome b5 might activate RTE1 through a redox modification, thus serving as the link between cellular redox status and ethylene signaling via the ETR1 ethylene receptor isoform. Alternatively, ETR1 conformation may be highly sensitive to changes in membrane composition and fluidity that are affected by the cytochrome-controlled unsaturated fatty acid content of membrane lipids (Chang et al. 2014). Cytochrome P450 helps form the exine layer of the outer pollen wall to produce normal mature pollen grains (Pinot and Beisson 2011). The cytochromes are commonly involved in the synthesis of secondary metabolites and the regulation of plant hormone metabolism. However, the role of the cytochromes encoded by DEGs identified in this study remains unclear.

#### Lipid and sugar metabolism

Plants require lipids for membrane biogenesis, as signal molecules, and as a form of stored carbon and energy (Schmid 2015). Lipids are present in membranes and influence cytoskeleton plasticity. Ethylene can influence lipids in the membranes (Jia and Li 2015). We identified DEGs (5.8%) related to lipid metabolism (Tables 8, S3, and S9). We speculate that in developing floral buds, the newly created male and female organs may be differentially sensitive to ethylene because of differences in lipid metabolism. Moreover, the synthesis of lipid components in anthers is essential for male reproductive development in plants, although the functions of these components have not been characterized. The lipid-based anther cuticle together with the pollen exine represents the two main protective barriers for microspores and pollen grains against various abiotic and biotic stresses (Zhang et al. 2010).

**Table 8:** Differentially expressed genes related to lipid metabolism

Comparison	Gene ID	Description	Specificity	Log2FC	Adj *p* value
Female FvM	*Cucsa.019600*	Bifunctional inhibitor/lipid-transfer protein	Lipid	− 1.63	2.16E−06
Female FvM	*Cucsa.320750*	Bifunctional inhibitor/lipid-transfer protein	Lipid	− 1.37	4.89E−05
Female FvM	*Cucsa.320770*	Bifunctional inhibitor/lipid-transfer protein	Lipid	− 2.73	1.73E−12
Female FvM	*Cucsa.096300*	Fatty acid desaturase 5	Lipid	− 2.72	6.75E−05
Female FvM	*Cucsa.250860*	PEBP (phosphatidylethanolamine-binding protein)	Lipid	− 2.92	5.81E−04
Female FvM	*Cucsa.091270*	lipoxygenase 1	Lipid	− 1.76	2.56E−07
Female FvM	*Cucsa.091280*	PLAT/LH2 domain-containing lipoxygenase	Lipid	− 1.72	2.56E−07
Herma MvH	*Cucsa.091380*	Lipoxygenase 1	Lipid	1.65	2.79E−03
Male FvM	*Cucsa.096340*	Fatty acid desaturase 5	Lipid	7.99	3.22E−16
Male FvM	*Cucsa.340370*	3-Ketoacyl-CoA synthase 7	Lipid	5.32	1.37E−14
Male FvM	*Cucsa.199000*	Bifunctional inhibitor/lipid-transfer protein	Lipid	4.66	7.62E−12
Male FvM	*Cucsa.257050*	Fatty acid hydroxylase superfamily	Lipid	1.11	5.89E−03
Male FvM	*Cucsa.257060*	Fatty acid hydroxylase superfamily	Lipid	2.70	2.40E−05
Male FvM	*Cucsa.304270*	GDSL-like Lipase/Acylhydrolase superfamily protein	Lipid	6.34	7.50E−03
Male FvM	*Cucsa.360940*	GDSL-like Lipase/Acylhydrolase superfamily protein	Lipid	2.39	9.14E−05
Male FvM	*Cucsa.054230*	Myzus persicae-induced lipase 1	Lipid	1.94	4.07E−02
Male FvM	*Cucsa.161220*	Polyketide cyclase/dehydrase and lipid transport	Lipid	2.78	1.51E−06
Male FvM	*Cucsa.372130*	Polyketide cyclase/dehydrase and lipid transport	Lipid	2.36	2.24E−06

Almost 7% of the identified DEGs were related to sugar metabolism (Tables 9, S3, and S9). Previous studies proved the involvement of the sugar signaling pathway during cucumber flower sex determination (Terefe and Tatlioglu 2005; Miao et al. 2010). The contents of sugars (e.g., sucrose and glucose) in the shoot apex are correlated with plant femaleness under different temperature conditions. The relevant functions of sugar signaling are unclear. The results of an earlier study (Miao et al. 2010) indicated that femaleness is enhanced by the accumulation of sugar in the shoot apices under low temperature. Additionally, sugars might influence ethylene signaling by controlling *Ethylene Response 1* expression (Sulmon et al. 2007). The accumulation of sugar induced by low temperatures upregulates *ACS2* expression, leading to ethylene biosynthesis and femaleness (Miao et al. 2010).

**Table 9:** Differentially expressed genes related to sugar metabolism

Comparison	Gene ID	Description	Specificity	Log2FC	Adj p-val
Female FvM	*Cucsa.334200*	Aldolase superfamily protein	Sugar	− 1.01	1.67E−02
Female FvM	*Cucsa.046350*	dormancy-associated protein-like 1	Sugar	− 0.93	1.54E−02
Female FvM	*Cucsa.089500*	Glucose-methanol-choline (GMC) oxidoreductase	Sugar	− 1.69	3.46E−02
Female FvM	*Cucsa.321550*	Haloacid dehalogenase-like hydrolase (HAD)	Sugar	− 1.81	1.51E−09
Female FvM	*Cucsa.109770*	polyol/monosaccharide transporter 5	Sugar	− 1.00	1.79E−02
Female FvM	*Cucsa.334200*	Aldolase superfamily protein	Sugar	− 1.01	1.67E−02
Herma FvH	*Cucsa.081220*	Galactose mutarotase-like superfamily protein	Sugar	Inf	7.63E−17
Herma FvH	*Cucsa.359700*	Glucose-methanol-choline (GMC) oxidoreductase	Sugar	3.09	1.72E−05
Herma MvH	*Cucsa.166390*	Phloem protein 2-A13	Sugar	1.40	2.85E−02
Male FvM	*Cucsa.256730*	Nucleotide-diphospho-sugar transferase	Sugar	5.83	1.89E−02
Male FvM	*Cucsa.254230*	Beta-galactosidase 7	Sugar	Inf	5.22E−04
Male FvM	*Cucsa.336940*	Beta glucosidase 11	Sugar	5.13	1.17E−19
Male FvM	*Cucsa.042020*	Beta-amylase 4	Sugar	2.12	7.71E−04
Male FvM	*Cucsa.109300*	Beta-galactosidase 3	Sugar	4.37	4.37E−06
Male FvM	*Cucsa.284640*	Glucuronidase 2	Sugar	4.20	8.48E−03
Male FvM	*Cucsa.154490*	Haloacid dehalogenase-like hydrolase (HAD)	Sugar	1.75	9.14E−05
Male FvM	*Cucsa.167470*	Lectin-related	Sugar	2.21	1.51E−02
Male FvM	*Cucsa.104560*	Myo-inositol-1-phosphate synthase 2	Sugar	0.98	2.40E−02
Male FvM	*Cucsa.332640*	Phosphofructokinase family protein	Sugar	2.37	1.46E−08
Male FvM	*Cucsa.349380*	Senescence-associated gene 29	Sugar	8.35	4.89E−02
Male FvM	*Cucsa.088480*	Sucrose-phosphate synthase family protein	Sugar	1.68	4.68E−04
Male FvM	*Cucsa.302770*	Sugar transporter 1	Sugar	Inf	2.30E−03
Male MvH	*Cucsa.340510*	Aldolase-type TIM barrel family protein	Sugar	− 1.88	2.76E−02

#### Ubiquitination processes

We identified an interesting group of genes that were related to the ubiquitin proteasome system (UPS), which degrades proteins. Some of the UPS-related genes (Tables S3 and S9) exhibited upregulated expression in females [RING proteins (*Cucsa.046340* and *Cucsa.132720*) and polyubiquitin protein (*Cucsa.266970*)] and in males [F-box proteins (*Cucsa.198720* and *Cucsa.351010*), BTB protein (broad-complex/tramtrack/bric-a-brac protein that recruits substrates to be degraded) (*Cucsa.103110*), and ubiquitin-like protein (*Cucsa.148380*)]. Ubiquitination involves the following three major enzymes: E1 activating enzyme, E2 conjugating enzyme, and E3 ubiquitin ligase. One of the best characterized RING ligases, the SCF (Skp–Cullin–F-box) complex, is composed of four major components, namely Skp1, Cul1/Cdc53, Roc1/Rbx1/Hrt1, and F-box protein (Ho et al. 2006). We identified a DEG encoding an F-box protein. The SCF/F-box-mediated ubiquitination processes provide control over the stability of proteins and play essential roles in many aspects of development. Both the E2 and E3 enzymes coordinate the transfer of ubiquitin onto a selected target and ultimately create a chain of ubiquitin molecules on the target. Ubiquitination can influence protein functions by directing cellular localization, modulating activation, and controlling abundance. Moreover, the UPS is crucial for the regulation of ethylene biosynthesis because it influences ACS synthase abundance and activity (Lyzenga and Stone 2012). This process may be affected by light intensity (Yoon and Kieber 2013). Interestingly, light also influences sex determination in cucumber flowers. We identified female-specific, light chain-related genes that exhibited upregulated expression in female cucumber lines (*Cucsa.067140* and *Cucsa.341570*). These genes may affect the development of floral bud sex types. There may also be an additional rate-limiting factor that mediates ethylene biosynthesis, with implications for phenotype development. Ubiquitination is also involved in PCD. There is currently no evidence that PCD occurs in cucumber floral buds, although some PCD-related phenomena have been observed in differentiating floral buds (Hao et al. 2003).

The data presented herein confirms that flower development involves the differential expression of vast number of genes. As many of DEGs determined in this study are known or predicted to have regulatory functions, it appears that the gene networks controlling floral organogenesis are very complex. Additional studies of selected genes are needed.

However, because they have minor and complex roles in flower formation, detailed functional characterizations via gene inactivation using modern techniques (e.g., genome editing with CRISPR/Cas9 or transformation) may be difficult because of a lack of a readily discernable sex mutant. The growing need to elucidate the functions of specific proteins and related interaction pathways in molecular networks is the driving force behind the development of analytical techniques (e.g., genome editing). It will soon be possible to efficiently generate full loss-of-function alleles and subsequently create lines with multiple genes knocked out. Thus, many additional regulators of flower development will likely be discovered in the near future, and this will lead to an increasingly comprehensive view of the gene sets that control floral organogenesis (Thomson et al. 2017). Even though transcripts correlated with sex expression in cucumber have been extensively studied (Guo et al. 2010; Przybecki et al. 2003, 2004; Wu et al. 2010) till now there is no clear view what is happening in developing floral buds.

Herein, we confirm that cucumber sex determination is a very complex process involving more metabolic pathways than previously thought (e.g., ethylene biosynthesis and other hormone-signaling pathways). Confirming the involvement of potential regulatory processes, such as ion homeostasis, cell wall and cytoskeleton modifications, ubiquitination, lipid and sugar metabolism, and gene expression mediated by transcription factors, represents an interesting challenge for researchers interested in gaining further insights into the cucumber sex determination process.

## Conclusions

High-quality transcriptome datasets for cucumber leaves and the shoot apex (with developing flower buds) were obtained using the Illumina HiSeq 2000 sequencing platform. A significant number of important metabolic pathways and functions associated with the unigenes were identified. Many candidate genes that are potentially involved in flower morphogenesis were applied to create a molecular network of interactions involving transcription factors, hormones, metal ions, cell wall and cytoskeleton modifications, transporters, cytochromes, sugars, lipids, and ubiquitination pathways. This molecular network may be useful for future studies regarding cucumber flower sex determination. Future studies involving more individuals from different genetic backgrounds grown under different environmental conditions are needed to assess the utility of specific transcripts as tissue- and/or sex-specific markers. Future investigations on the regulation of our candidate genes may enable researchers and breeders to control or select specific sexual expression patterns in cucumber. The functional annotation of high-throughput transcriptomic data generated in this study may serve as genetic resources for the development of crop improvement strategies for cucumber and other related crops.

### Author contribution statement

MP, ZP, and RKW designed and conceived the experiments. MP, AS, and LP completed the experiments. MP, LP, AS, JR, and KP analyzed the data. MP and WP wrote the manuscript.

## Electronic supplementary material

Below is the link to the electronic supplementary material.

Supplementary material 1 (XLSX 11 kb)

Supplementary material 2 (XLSX 2196 kb)

Supplementary material 3 (XLSX 313 kb)

Supplementary material 4 (XLSX 11 kb)

Supplementary material 5 (XLSX 38 kb)

Supplementary material 6 (XLSX 12 kb)

Supplementary material 7 (XLS 232 kb)

Supplementary material 8 (XLSX 116 kb)

Supplementary material 9 (XLS 94 kb)
